# Free-standing, thin-film sensors for the trace detection of explosives

**DOI:** 10.1038/s41598-021-86077-6

**Published:** 2021-03-23

**Authors:** Peter P. Ricci, Otto J. Gregory

**Affiliations:** Sensors and Surface Technology Partnership, Department of Chemical Engineering, University of Rhode Island, 2 East Alumni Avenue Suite 360, Kingston, RI 02881 USA

**Keywords:** Chemical engineering, Sensors and biosensors

## Abstract

In a world focused on the development of cybersecurity, many densely populated areas and transportation hubs are still susceptible to terrorist attacks via improvised explosive devices (IEDs). These devices frequently employ a combination of peroxide based explosives as well as nitramines, nitrates, and nitroaromatics. Detection of these explosives can be challenging due to varying chemical composition and the extremely low vapor pressures exhibited by some explosive compounds. No electronic trace detection system currently exists that is capable of continuously monitoring both peroxide based explosives and certain nitrogen based explosives, or their precursors, in the vapor phase. Recently, we developed a thermodynamic sensor that can detect a multitude of explosives in the vapor phase at the parts-per-trillion (ppt) level. The sensors rely on the catalytic decomposition of the explosive and specific oxidation–reduction reactions between the energetic molecule and metal oxide catalyst; i.e. the heat effects associated with catalytic decomposition and redox reactions between the decomposition products and catalyst are measured. Improved sensor response and selectivity were achieved by fabricating free-standing, ultrathin film (1 µm thick) microheater sensors for this purpose. The fabrication method used here relies on the interdiffusion mechanics between a copper (Cu) adhesion layer and the palladium (Pd) microheater sensor. A detailed description of the fabrication process to produce a free-standing 1 µm thick sensor is presented.

## Introduction

The detection of low-volatility plastic explosives as well as peroxide based explosives in many densely populated areas and transportation hubs continues to be a priority for those tasked with protecting the public from such threats. Many of these explosives have been employed by terrorists in improvise explosives devices (IEDs) as both initiators and detonators. Over the last decade, IEDs employing these explosives have been responsible for a variety of terrorist attacks around the world, including, the March 2016 Brussels bombing. These explosives can be peroxide-based or nitrogen-based depending on their chemical composition. Most explosive detection systems are tuned for certain nitrogen-based compounds, making detection of many peroxide based explosives, such as triacetone triperoxide (TATP), increasingly challenging. Meanwhile, some nitramines including Research Department eXplosive (RDX), High Melting Explosive (HMX), etc. possess relatively low vapor pressures making detection in the vapor phase extremely difficult^1^. Despite these low vapor pressures, most explosives, decompose and/or sublime. Therefore, these explosives exist in the vapor phase at trace levels providing the impetus for a continuous monitoring platform.

Currently, densely populated venues and transportation hubs are patrolled by canines which are trained to selectively identify peroxide and nitrogen based IEDs^2,3^. By virtue of their highly sensitive noses, these dogs are capable of detecting explosives at remarkably low levels (< ppt) and employ directionality which leads to precise identification of an IED’s location^2^. However, training these bomb sniffing dogs is expensive and can take multiple months thus limiting their availability. Additionally, these dogs are limited in their capability in that they can lose their effectiveness after several hours and require periodic rest. Detection of solid, liquid, and vapor phase explosives has also been displayed by several other platforms employing a variety of detection mechanisms. For example, fluorescent techniques have been used for the detection of peroxide-based explosives and nitramines in the liquid and vapor phase respectively. These techniques employ a variety of fluorescent enabled materials^4–7^ which are quenched in the presence of the explosive analyte. However, peroxide-based explosives (such as TATP) do not exhibit fluorescence quenching in the vapor phase^8^ and thus require preconcentration in standard solutions for liquid phase detection. Additionally, florescent detection of nitramine compounds has only been demonstrated in the vapor phase at increased concentrations (ppm) which limits its functionality. Meanwhile, fluorescent detection of nitrite compounds has been displayed with impressive selectivity but has so far been limited to the liquid phase^7^.

Trace detection of both nitrogen and peroxide based explosives has also been addressed using a variety of lab-scale analytical techniques^9–19^. Gas chromatography and mass spectroscopy^9–14^ are capable of selectively identifying trace explosive molecules within a single sample despite the presence of interfering background gases. These techniques are modular in that they can even be used to sample ambient environments using atmospheric flow tubes^11^. Unfortunately, these systems possess a large footprint relative to small-scale chemical sensors which limits their feasibility and functionality in a portable platform. Other analytical methods such as, ion mobility spectroscopy (IMS)^15–19^ and liquid chromatography^20^, require pretreatment of the analyte prior to analysis which severely increases the time required for sample processing and thus, cannot be used for continuous, real-time detection. Colorimetric^19,21–24^ and voltametric^25^ sensors have shown promising detection of peroxide based explosives and nitrates at trace levels. However, both sensor platforms are limited due to their relatively slow response times and often require aqueous phase detection. Colorimetric olfactory sensor arrays have mitigated many of these limitations and displayed improved selectivity but have yet to be demonstrated for the detection of many peroxide and nitrogen based explosives^24^. Trace detection of TATP has been displayed by a potentiostatic sensors with titania nanotubes^26^ but were somewhat limited in traditional humid/ambient conditions.

As previously described by Rossi et al.^27^, our thermodynamic sensor relies on two separate microheaters. The first microheater is coated with one of several different metal oxides (catalysts) which are used for the detection of vapor phase analytes. The second microheater is a reference, which is left uncoated and is used to subtract any sensible heat effects (and hydrodynamic effects) from the active sensor response that are not associated with the catalyst/analyte interaction. The reference signal is subtracted from the catalyst-coated signal and thus, the heat effect due to catalytic decomposition alone is measured. This effectively mitigates false positives and negatives. By virtue of the catalyst coated sensor, the thermodynamic platform also measures the heat effect associated with oxidation/reduction reactions. For explosives, the vapor phase molecule catalytically decomposes into known decomposition products upon interaction with the catalyst surface which in turn interact with the catalyst causing specific redox reactions^27^. The catalytic decomposition and subsequent redox reactions release heat energy that is accurately measured using feedback/control circuitry. This heat energy results in an electrical power difference required to maintain both microheaters at the same temperature. In the case of TATP, acetone and hydrogen peroxide are produced as the known decomposition products upon interaction with the catalyst surface. As mentioned above, these products also interact with catalyst resulting in specific redox reactions, which in turn effect the oxidation state of the catalyst^28^. In general, oxidation reactions release heat resulting in less electrical power required to maintain the same temperature and thus negative responses. Conversely, reduction reactions absorb heat requiring more electrical power and thus positive responses. For explosives, heat effects produced through redox reactions greatly exceed those produced by the catalytic decomposition of the analyte molecules and dominate the sensor response^28^.

These catalytic decomposition/redox reactions can be exothermic or endothermic depending on three factors: the analyte, the metal oxide catalyst, and the operating temperature of the sensor^28^. In the case of a tin oxide catalyst (SnO^1+^), redox reactions dominate the response of the sensor at temperatures greater than 75 °C and produce large heat effects. At temperatures less than 75 °C, the oxidation state of SnO^1+^ is unaffected by the presence of the decomposition products producing responses based solely on catalytic decomposition. In this way, a single catalyst can be used for orthogonal detection of vapor phase analytes based on its temperature set-point.

A reduction in thermal mass of the sensing platform was accomplished by utilizing 20 µm thick yttria-stabilized zirconia (YSZ) ribbons as the substrate for the thin film microheaters^28^. This not only enabled greater sensor response but also lowered the power requirements to operate the sensors, which makes the sensor portable enough to be deployed on drones and wearables. Further improved sensor response was accomplished by fabricating free-standing, thin-film microheaters, which represents the lowest theoretical thermal mass for this type of sensor platform. To accomplish this, the microheater serpentine was completely removed from the substrate surface resulting in a sensor with a nominal thickness of ~ 1 µm. In comparison to previous versions of our sensor, the free-standing sensor displayed unparalleled sensor response and selectivity to a variety of vapor phase analytes and enabled the real-time detection of explosives and their precursors.

Other groups have successfully employed microheater based gas sensors for the detection of vapor phase analytes^29–35^. These sensors are fabricated using traditional techniques including standard sputter deposition and etch-back for MEMS based platforms^29^. These microelectromechanical systems (MEMS) display minimal power requirements (~ 30–40 mW) to reach desired operating temperatures (100–500 °C) and could be modified with nanowires^30^ or reduced graphene oxide hydrogels^31^ for improved sensor response. Similarly, field effect transistors (FETs) with embed microheaters have shown the ability to detect NO_2_ at the high part-per-billion (ppb) level with incredible power efficiency (1.63 mW at 300 °C)^32^. Unfortunately, the fabrication process for these systems can be relatively complex and require time and resources to replicate thus limiting their availability. Interestingly, tin oxide microheaters employing tin oxide sensing materials have been fabricated that displayed remarkably fast response and recovery times (~ 10 s respectively) for the detection of methane^34^. Ultimately, these sensors required extremely high operating temperatures > 700 °C for optimal performance. ZnO modified MEMS have comparatively higher operating powers (386 mW at 350 °C) but have displayed the ability to detect NO gas as low as 50 parts-per-billion (ppb)^35^. Our free-standing, thin-film microheaters represent a balance between power consumption and sensor response. These sensors display power consumption levels greater than most MEMs platforms (~ 150 mW at 175 °C) but display remarkable sensor responses at significantly lower concentrations (< ppt) than the platforms described above. As described below these microheaters are also extremely stable and reproducible under ambient conditions, having survived hundreds of cycles with very little signal variation. Additionally, our novel fabrication process relies on simple properties inherent to the microheater materials themselves, promoting easy and rapid production of free-standing, thin-films. This straightforward fabrication process and impressive sensor performance are key advantages over traditional microfabrication processes. A detailed description of the sensor fabrication and response are described below.

## Results and discussion

### Free-standing sensor fabrication

**Figure 1:**
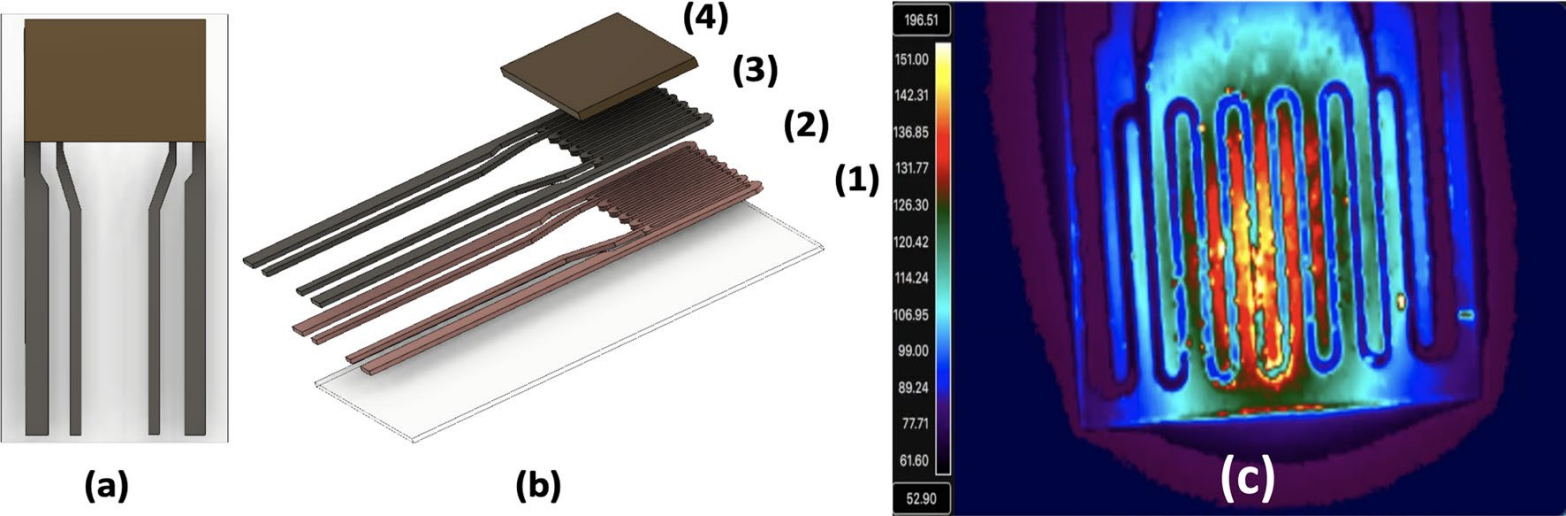
Schematic of a thermodynamic sensor (**a**), (**b**) showing the various layers comprising the sensor: YSZ substrate (1), copper adhesion layer (2), palladium microheater (3), metal oxide catalyst (4) and a high resolution IR image of a thin film microheater fabricated onto a 20 µm thick YSZ substrate responding to an explosive analyte (**c**).

The thermodynamic sensor was constructed on ultrathin, 20 µm thick yttria-stabilized zirconia (YSZ) ribbons measuring 1.6 cm × 0.7 cm. Photolithography techniques were used to pattern ~ 1 µm palladium microheaters onto the YSZ substrates. Prior to palladium deposition, a 400 Å thick layer of copper was sputter-deposited onto the substrate, which acted as an adhesion layer between the microheater and the YSZ. Each microheater had four leadouts, which provided communication between the sensor and the digital control system. A schematic of the various layers comprising the thermodynamic sensor is shown in Fig. 1. Here, noble metals such as Pd and Pt displayed poor adhesion to the ultrathin YSZ, thus a variety of other metals including nickel, chrome, copper, and titanium were investigated as adhesion promoters. Of these, copper was determined to yield the most adherent films to the YSZ substrate. As previously described in Ricci et al.^28^, palladium is used as our microheater metal due to its inherent catalytic properties and minimal oxide formation. The Cu/Pd system also displays simple diffusion at relatively low temperatures which plays an important role in the fabrication process described below.

**Figure 2:**
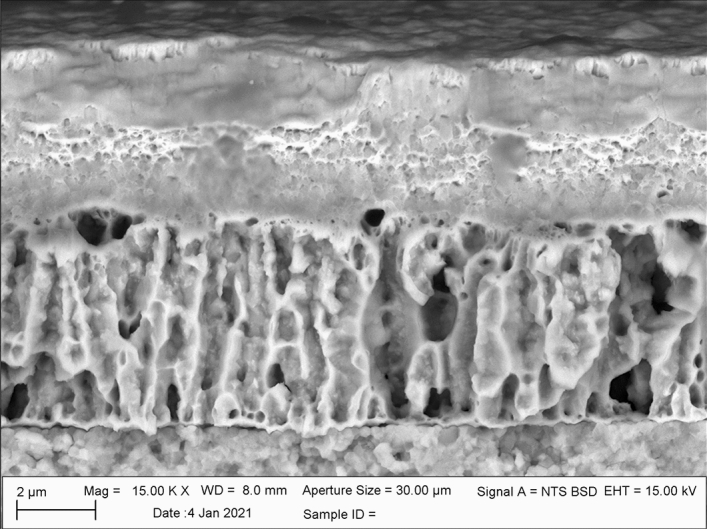
SEM micrograph of a 3 µm thick Pd film deposited onto a 5 µm thick Cu film (as-deposited condition).

Fabrication of free-standing, thin film sensors has been demonstrated using a variety of methods^36–39^. Similar to the MEMs systems described above, many of these fabrication processes are complex and often required considerable time and resources to replicate. The fabrication of our free-standing (microheater) sensors relies on the selective interdiffusion between the Cu and the Pd films. As mentioned above, Pd films display poor adhesion to the YSZ substrate. Thus, selective removal of the Cu adhesion layer would promote the formation of a free-standing thin-film without limiting the structural integrity of the sensor thin film leadouts, which remain firmly intact; i.e. they remain strongly bonded to the YZS substrate while only the serpentine lifted off. In order to establish the conditions to promote complete interdiffusion of the Cu into the Pd and delamination from the YSZ substrate, a 3 µm thick Pd film was deposited onto a 5 µm thick Cu film by rf sputtering. The 20 µm thick YSZ substrates were easily cleaved for cross-sectional analysis using scanning electron microscopy (SEM). Figure 2 shows an SEM micrograph of the cross section of a 3 µm thick Pd film deposited onto a 5 µm thick Cu film. By virtue of its lower atomic mass, the Cu film appeared slightly darker than the Pd film Additionally, the Cu thin-film exhibited a traditional columnar grain structure which was characterized by non-equiaxed grains growing normal to the surface; i.e. highly anisotropic growth was observed in the Cu layer (z-direction) and is consistent with the Thornton Zone II model^40^ for the normalized argon partial pressures and normalized temperatures employed for Cu depositions. However, the Pd films exhibited a largely amorphous structure consistent with a Thornton Zone I model, which lacked any distinct microstructural features. This structure is consistent with the Thornton Zone I model due to the elevated melting temperatures of Pd relative to Cu (1555 °C vs 1050 °C). These microstructural differences produced a visibly distinct boundary between the two metals which remained continuous across the “through thickness” of the films. Energy dispersive X-ray spectroscopy (EDS) was utilized to confirm composition of each film prior to heat treatment. As expected, each discrete film showed no evidence of interdiffusion between the Pd and Cu layers prior to annealing (Supplemental Material I).

**Figure 3:**
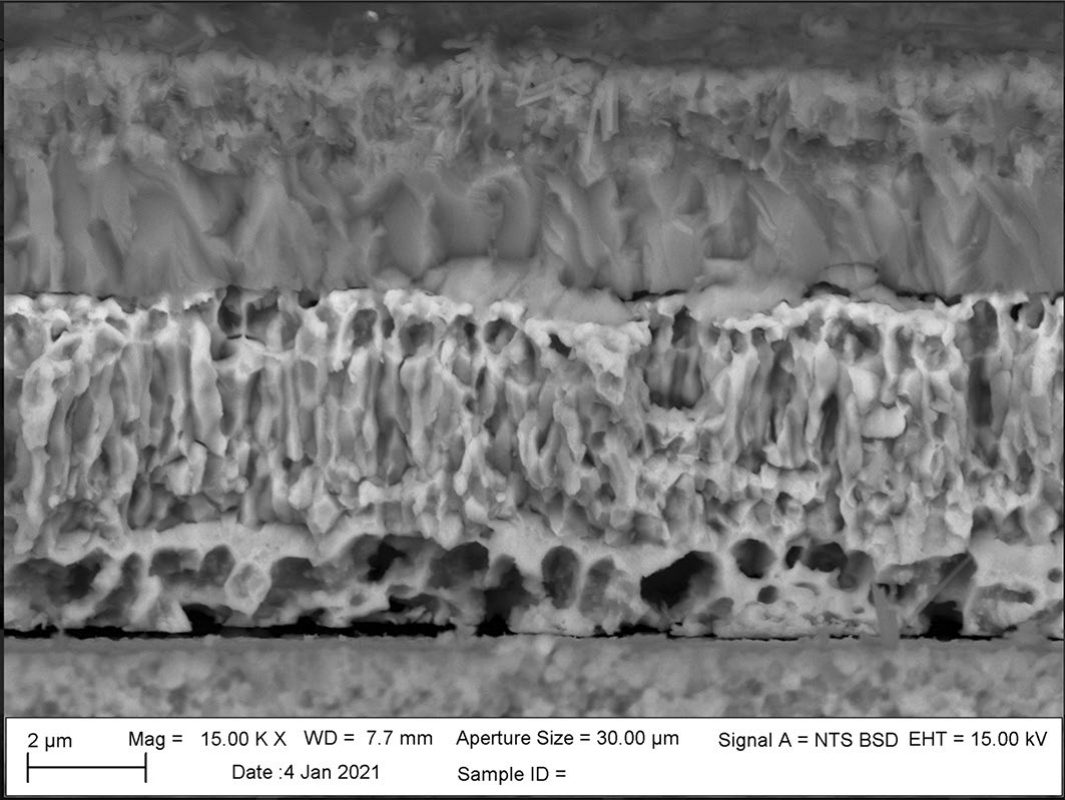
SEM micrograph of a 3 µm thick Pd film deposited onto a 5 µm thick Cu film after heating to 350 °C.

To promote interdiffusion, the Cu and Pd films were heated to 350 °C in a Mellen tube furnace (high purity alumina tube). The samples were then allowed to cool over the period of 10 h to insure complete diffusion. Figure 3 shows an SEM micrograph of Pd and Cu films deposited on a YSZ substrate and heated to 350 °C. Here, an SEM cross-section of the films indicated considerable interdiffusion of Cu. First, the distinct columnar grain structure associated with the Cu film transitioned to a more equiaxed grain structure. This columnar-to-equiaxed transition (CET) is an early indication of recrystallization, which occurs as a function of the temperature gradients formed during the heat treatment cycle^41^. These grains appear more irregular than the ordered columnar grains displayed in the as-sputtered condition. There are also changes in contrast between the two films indicating some interdiffusion. Figure 3, for example, shows less contrast between the two films than the as-deposited films in Fig. 2, which suggests some interdiffusion of the Cu. The presence of Cu within the Pd film was confirmed using EDS (Supplemental Material II).

**Figure 4:**
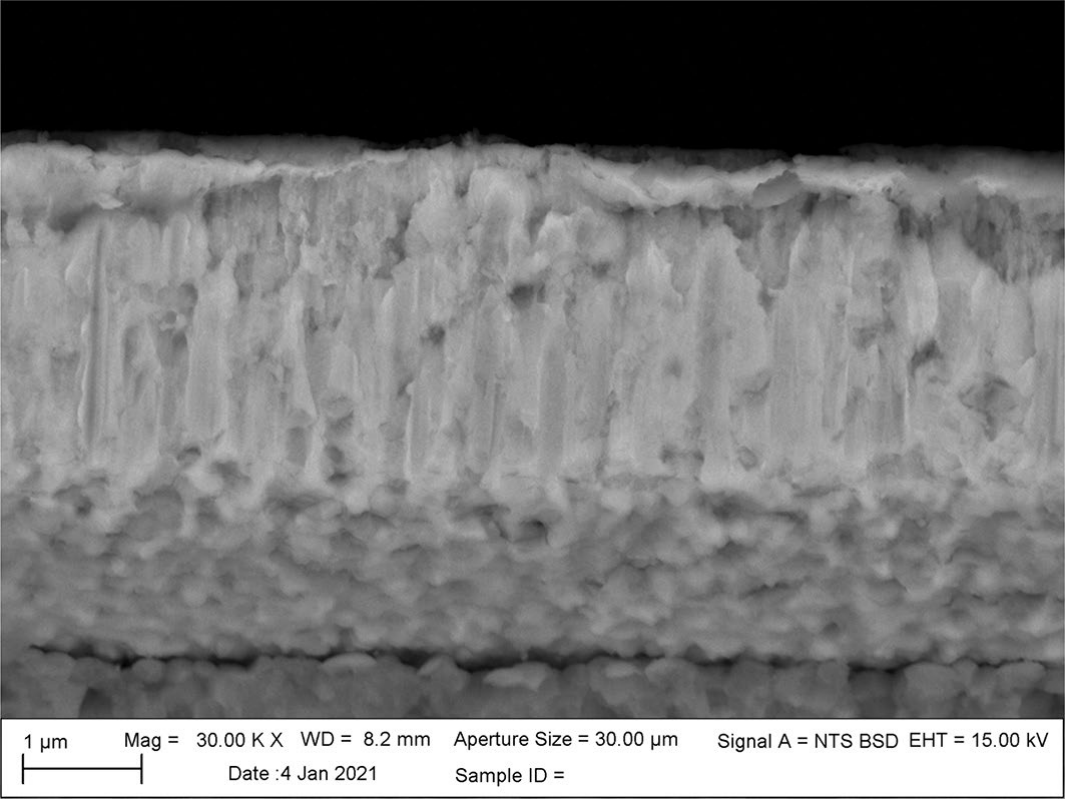
SEM micrograph of a 3 µm thick Pd film deposited onto a 5 µm thick Cu film after heating to 500 °C.

Interdiffusion of Cu into the Pd films was complete after heating to 500 °C for an hour and furnace cooling to room temperature. Figure 4 shows an SEM micrograph the Pd and Cu films on a YSZ substrate after heat treatment at 500 °C. Here, the SEM micrograph shows no distinct interface between the Cu and Pd films and the two films were replaced by a single amorphous film. This Cu/Pd “alloy” exhibited some striations which suggests that some segregation due to the formation of intermetallic may have occurred, which is consistent with the phase diagram for the system Cu–Pd^42^. The bright areas in Fig. 4 are Pd rich and the darker regions Cu rich, since the SEM imaging was done in backscattered mode. Interestingly, the samples showed the formation of a thin dark layer on the surface of the thin film, which was determined to be copper oxide (CuO). This CuO layer formed on the surface during heat treatment, even though heat treatment was done in a nitrogen ambient. Enough oxygen was present to oxidize copper reaching the outer surface and resulted in CuO formation. This provided the driving force for the continued removal of copper from the Pd film via oxidation. Under these conditions, the Cu continues to react with oxygen, depleting Cu in the Pd near the surface, which caused more Cu removal from the Pd film. Ultimately, the removal of the copper film was responsible for the delamination of the serpentine portion of the microheater. The lack of any copper at the YSZ/Pd interface and the presence of a native CuO film were confirmed using EDS (Supplemental Material III).

The fabrication of the free-standing sensors relied on the selective interdiffusion of Cu in the adhesion layer into the Pd film. Selective heating of the microheater (serpentine) at 500 °C would be difficult without affecting the integrity of the interface between the sensor leadouts and the YSZ. This selective heating was only achievable due to the intrinsic thermal properties of the ultrathin YSZ substrates. They exhibit highly localized heating in the “z” direction (Fig. 1c) and thus, the thermal properties are highly anisotropic with an in-plane thermal conductivity of 2.7 W/mK. This allows the heat to remain in the center of the serpentine without dissipating laterally to the rest of the sensor substrate. Thus, the metallization in the serpentine can be heated to 500 °C, while the leadout metallization remains at room temperature. This was accomplished by selectively heating the serpentine with a proprietary digital control system. Here, the free-standing microheater sensor was heated for 10 min at temperatures ranging from 50 to 500 °C. The exact temperature of the microheater was based on the temperature coefficient of resistance (TCR) and the resting resistance of the palladium microheater at room temperature^28^. After each heating cycle, the microheater was imaged and the resistance recorded in order to track the synthesis of the free-standing sensor serpentine.

**Figure 5:**
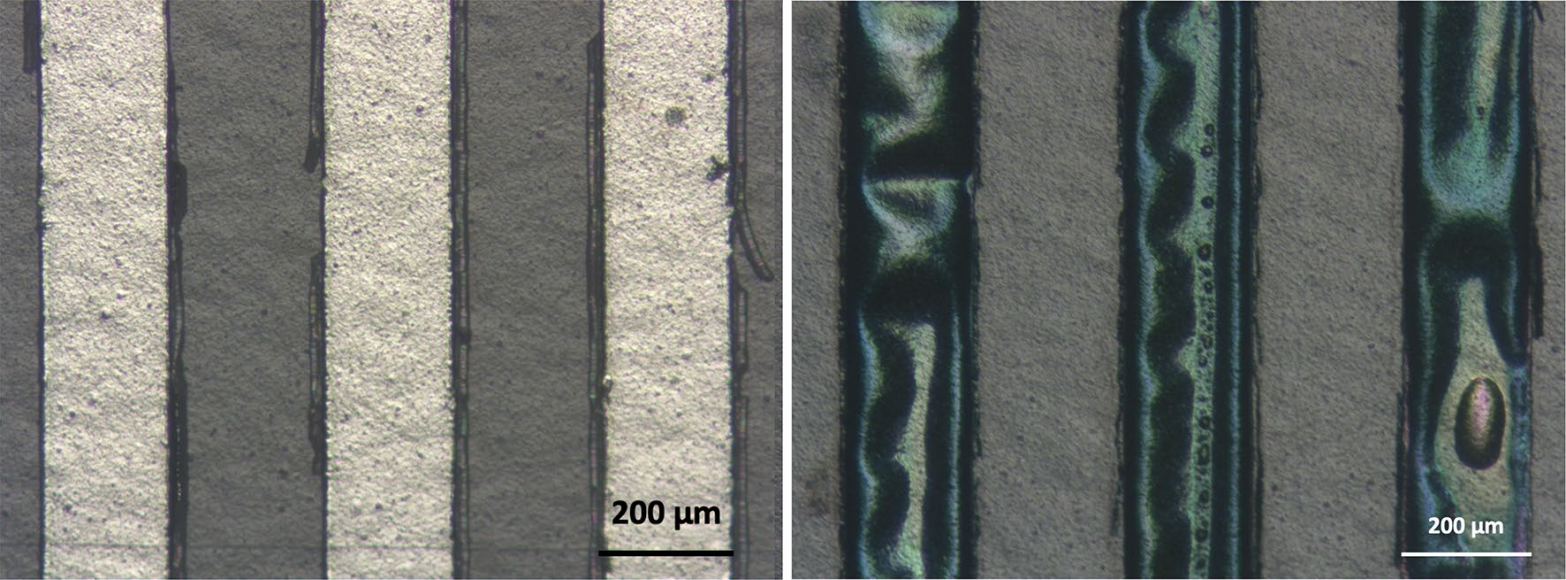
Optical micrographs of serpentine region of thin film microheater before (left) and after (right) heat treatment at 350 °C.

Prior to heat treatment, the resting resistance of the microheater was 33.3Ω at room temperature and the corresponding micrograph of the serpentine is shown in Fig. 5. The microheater was then heated to temperatures ranging from 50 to 200 °C. As expected, these heating cycles produced very little change in the appearance of the serpentine. The microheater was then heated to 350 °C for 10 min using the same procedure and an optical micrograph of the heat treated serpentine is shown in Fig. 5. At this temperature, it was obvious that Cu had diffused into the Pd and large segments of the Pd film delaminated from the YSZ substrate. Coalescence of microbubbles along the length of the serpentine were also observed in Fig. 5. As previously mentioned, the delamination of the metal film from the substrate suggests that selective removal of the copper adhesion layer had occurred. In some instances, microbubbles formed towards the center of the serpentine suggesting that coalescence of argon trapped in the films produced microbubbles that coalesced and promoted delamination as well. After heat treatment, the resistance of the microheater was 23.2Ω, which represents a 10.1Ω decrease over the baseline resistance. This resistance change was attributed to the release of argon trapped within the film as well as the reduction in point defects as result of sputtering, which lowered the resistance of the palladium film.

**Figure 6:**
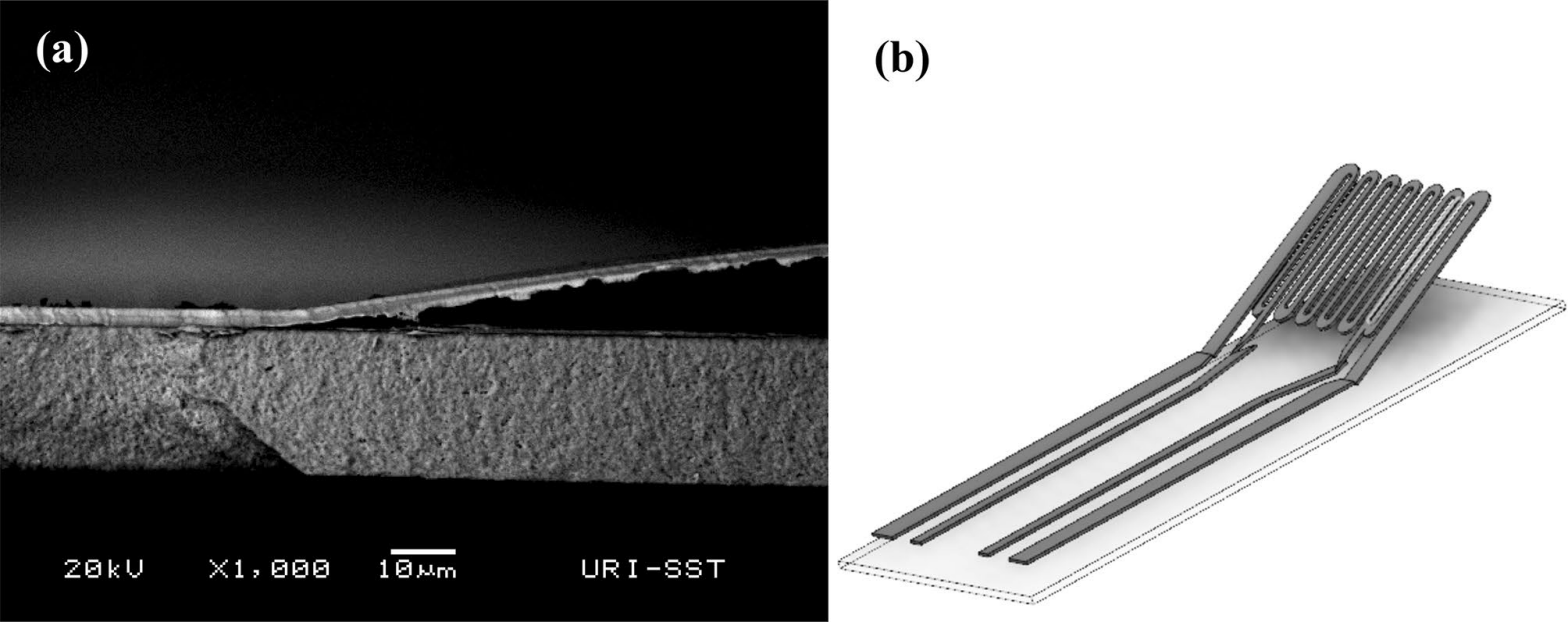
SEM micrograph of a free-standing Pd microheater after heat treatment at 500 °C (**a**). Schematic of the free-standing sensor showing delamination of the serpentine region of the microheater (**b**).

Lastly, the Pd/Cu microheaters were heat treated at 500 °C for 10 min to produce free-standing microheaters. At this temperature, the heat pattern shown in Fig. 1c expanded to edges of the microheater serpentine promoting complete lift-off without effecting the lower leadouts. Figure 6a shows an SEM image of a free-standing microheater that delaminated from the YSZ substrate after heat treatment at 500 °C. Here, the serpentine portion of the microheater was completely separated from the substrate while the thin film leadouts remained perfectly intact and strongly bonded to the YSZ substrate. A schematic of the free-standing sensor displaying delamination of the microheater serpentine is shown in Fig. 6b. This robust construction allows for easy manipulation of the sensor platform without risking damage to the free-standing thin-film. After fabrication of the free-standing thin-films, one of the microheaters was sputter deposited with a coating of SnO^1+^ catalyst over the microheater serpentine (Fig. 1a,b) while the other was left uncoated to act as a reference. The surface level CuO layer that was natively grown during the fabrication process was shown to be critically thin and thus unresponsive to the analyte (Supplemental Material IV-Fig. S1).

### Free-standing sensor response

**Figure 7:**
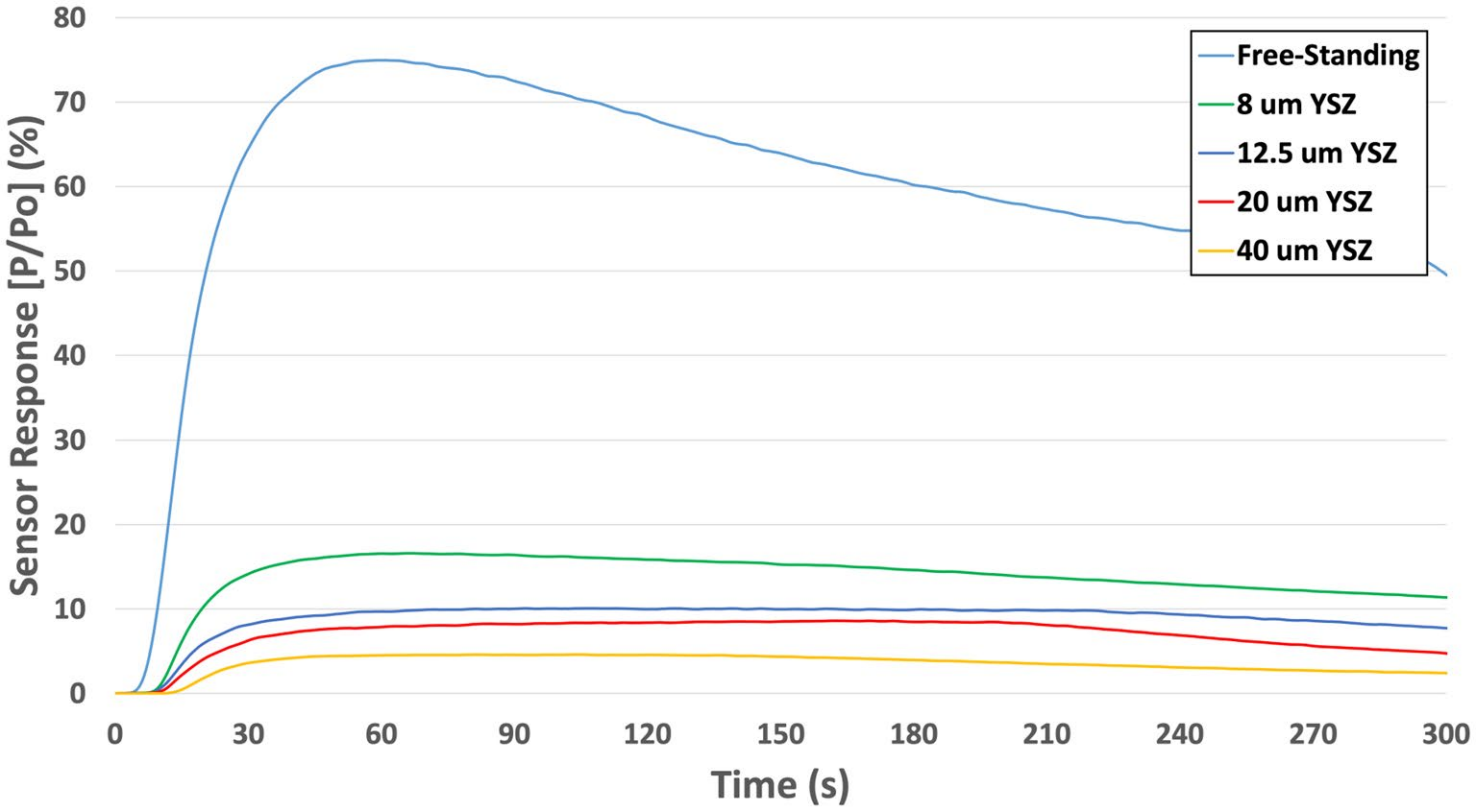
Responses of four different microheater sensors to 20 ppm TATP fabricated on YSZ substrates of varying thickness relative to the response of a free-standing microheater.

The sensors were exposed to a variety of vapor phase analytes including peroxide-based explosives (TATP, diacetone diperoxide (DADP) as well as nitramines, nitrates, nitroaromatics and their responses recorded. The testing was done according to the procedure described in the “Methods” section below. As mentioned, by Ricci et al.^28^, minimization of thermal mass by using ultrathin, low mass YSZ substrates led to significantly improved sensor response and selectivity with this type of sensor platform. Figure 7 shows the responses of four different microheater sensors to 20 parts-per-million (ppm) TATP when fabricated on YSZ substrates of varying thickness. In Fig. 7, the responses were also compared to the free-standing microheater sensor. Until now, 8 µm thick YSZ-based sensors displayed the highest sensor response (16%). However, the free-standing microheater showed considerable improvement in sensor response; e.g. 75% response to 20 ppm TATP and was attributed to the relatively small thermal mass of the free-standing sensor. The overall thickness of the sensor (~ 2 µm) forced the heat to remain along the catalyst surface with minimal dissipation of heat to the sensor platform. This isolated heating effect promoted more activation of the catalyst surface and improved overall sensor performance. The reduction in thermal mass resulted in dramatically lower power requirement as well. At an operating temperature of 175 °C, the free-standing sensor required only 150 mW, which represents a significant decrease over the 8 µm thick platform which required 250 mW to reach the same temperature. Much faster response times were observed with the free-standing sensor; e.g. the free-standing sensor required just 1 s to reach 10 percent of the maximum response (t10 response time) and only 30 s to reach 90 percent of the maximum response (t90 response time).

**Figure 8:**
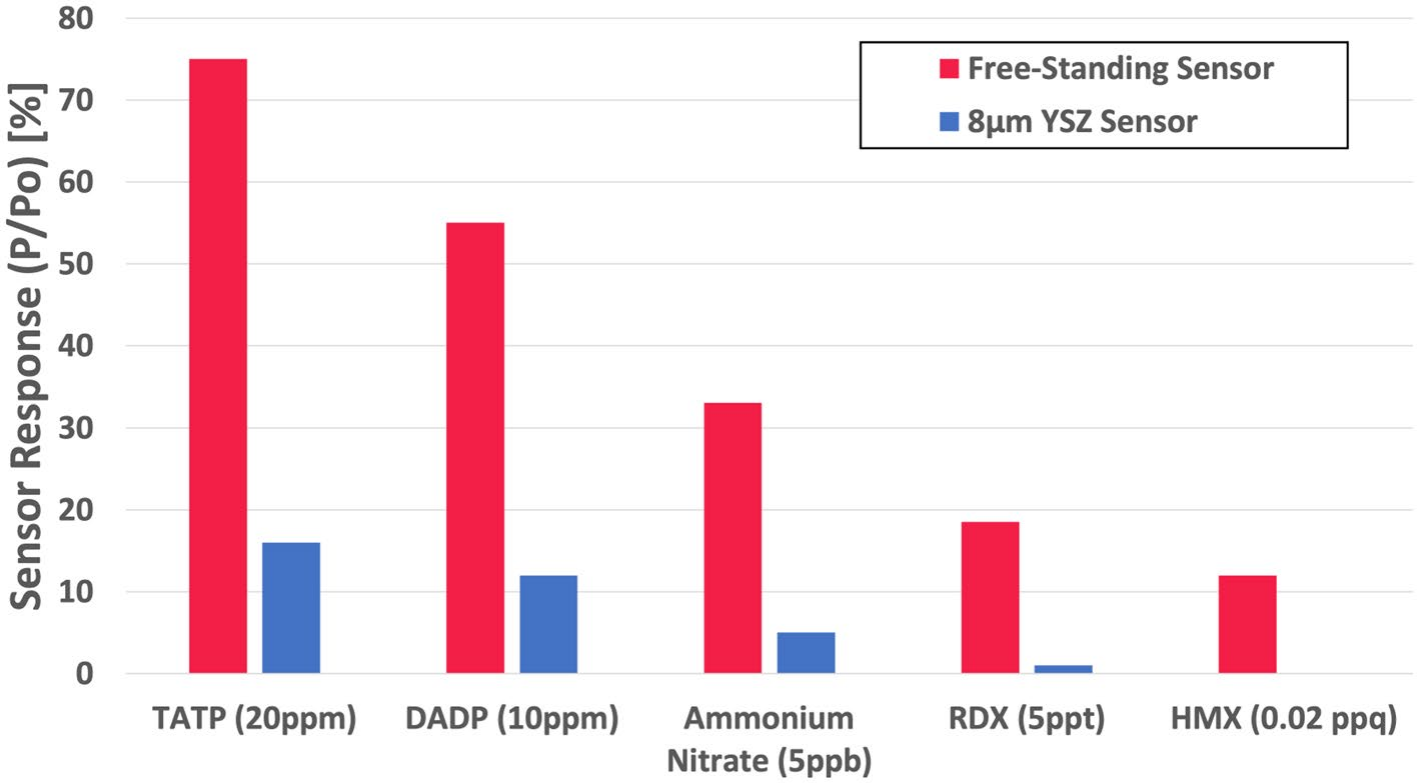
Responses of an 8 µm YSZ sensor and free-standing sensor to a variety of peroxide based explosives as well as nitramines and nitrites at known concentrations.

In order to further validate the improved performance of the free-standing sensor platform, the sensor was exposed to a variety of explosives over a range of known concentrations (Fig. 8). These explosives consisted of both peroxide-based explosives (TATP and DADP) as well as nitramines (RDX, HMX) and nitrates (ammonium nitrate (AN)). These compounds were specifically chosen due to their known vapor pressures^1^, enabling a wide range of concentrations to be investigated (20 ppm to 0.02 parts-per-quadrillion (ppq)). Here, the free-standing sensor outperformed the 8 µm YSZ sensor for all explosives tested. As expected, sensor performance coincided well with the known concentration of each explosive. Figure 8 also shows that the free-standing platform displayed the ability to detect low-volatility plastic explosive as well (See Supplemental Material IV-Fig. S2a,b for original test data). Specifically, the detection of RDX and HMX was achieved at concentrations at the 5 ppt and 0.02 ppq levels, respectively. These concentrations are considerably lower than most other explosive compounds due to their low vapor pressures, which further validates the ability of free-standing sensor platform to detect almost any explosive in the vapor phase.

**Figure 9:**
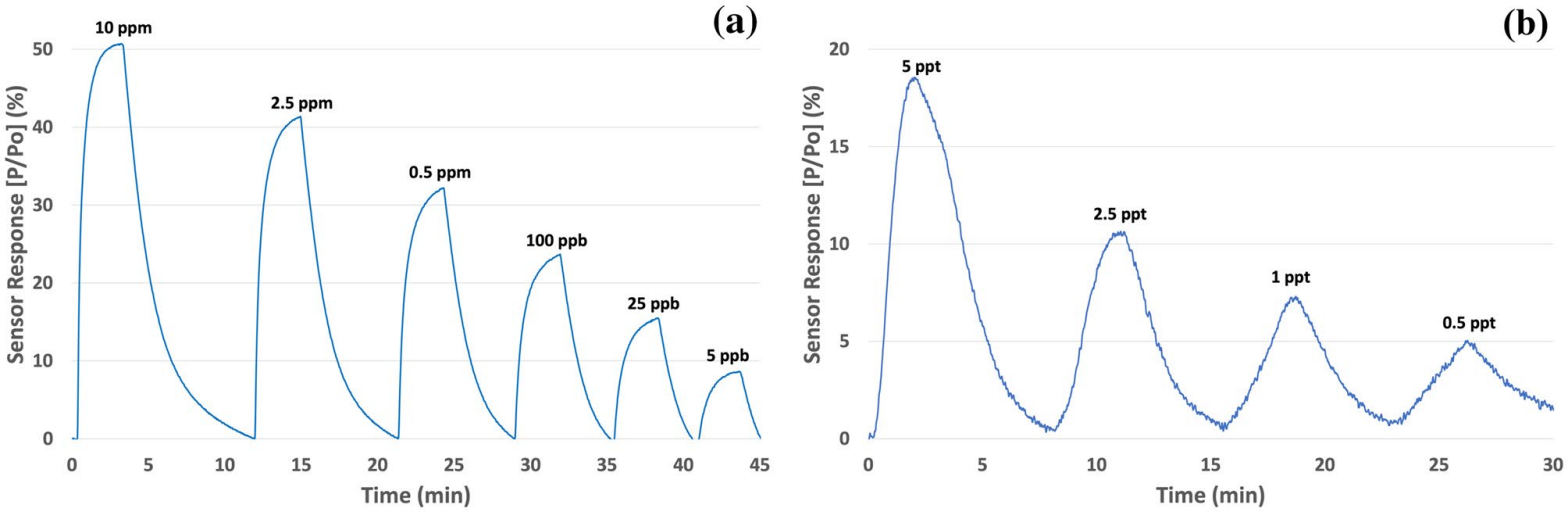
Response of a free-standing sensor to concentrations of TATP ranging from 5 ppb to 10 ppm (**a**) and concentrations of RDX ranging from 0.5 to 5 ppt (**b**).

In addition to sensor performance, validation of the sensor stability and reproducibility were investigated. Figure 9 shows the response of a free-standing sensor to concentrations of TATP ranging from 5 ppb to 10 ppm (a) and concentrations of RDX ranging from 0.5 to 5 ppt (b). For each explosive, the concentrations were introduced sequentially without powering off the sensor to ensure reproducible detection while maintaining sensor stability. The results showed that the sensor detected TATP and RDX over a range of concentrations without much variation in baseline power; i.e., the response curves reliably returned to zero after the detection at each subsequent concentration. As expected, the free-standing sensor displayed decreasing sensor response which correlated well with the decreasing concentrations. These experiments were repeated over multiple cycles in order to ensure consistent reproducibility.

The free-standing sensor also showed relatively fast response times despite decreasing concentrations. Figure 9b shows that the t10 response time increased from 26 to 43 s for detection of RDX at concentrations of 5 ppt and 0.5 ppt respectively. Similarly, the t90 response time increased from 1.5 to 2.6 min for the detection of RDX at the same two concentrations. The current testing apparatus delivers flowrates of approximately 100SCCM which limits the mass transport of analyte to the sensor’s active catalytic sites. Increased flow rates will promote faster saturation of the catalyst active sites and thus improved response times. Despite this relatively slow flowrate, the results shown in Fig. 9b represent an order of magnitude decrease in concentration with only a < 50% increase in response time. During field operation, the overall duty cycle (response to full recovery) can be reduced by rapid cycling of the sensor platform. Currently, the overall duty cycle is > 5 min due the time dependent mass transport of analyte from the sensor’s catalytic sites during recovery. In order to minimize this duty cycle, the sensor’s catalyst layer can be “shocked” through rapid thermal cycling post detection. The relatively minimal thermal mass of the free-standing sensors allows them to be cooled to room temperature in seconds after which they can be quickly reheated. Despite the fragile structure of the free-standing sensor serpentine, they have survived hundreds of these cycles without failure and were resilient to humidity effects (Supplemental Material IV-Fig. S3a,b). During field operation, this procedure will promote truly continuous detection in real-time at extremely low concentrations. Additionally, the sensors displayed < 3% signal variation at each condition which means the sensor measurement can be both qualitative and quantitative as required.

## Conclusions

Utilizing the interdiffusion characteristics in the Cu/Pd system and the highly anisotropic thermal conductivity of ultrathin yttria-stabilized zirconia substrates, a manufacturable fabrication sequence for producing robust free-standing thin-film sensors for trace detection of explosives was demonstrated. Microanalysis suggested that interdiffusion of Cu into Pd was complete at temperatures approaching 500 °C. Initially, the diffusion of Cu into Pd led to a transition from a columnar microstructure to an equiaxed microstructure before forming a single-phase Cu/Pd “alloy.” This lift-off technique was applied to the microheaters by heating just the serpentine and allowing it to be completely separated from the substrate without sacrificing the structural integrity of the thin film leadouts bonded to the substrate. These free-standing thin film sensors yielded superior performance in terms of sensor response and response time by minimizing thermal mass. This enabled the detection of a variety of peroxide based explosives as well as nitroaromatics and nitrates at the ppt level or better. Most notably, the detection of plastic explosives such as RDX and HMX was achieved at concentrations of 0.5 ppt and 0.02 ppq. The reduction in thermal mass also significantly lowered the power requirements for the sensors to < 175 mW. These sensors have also displayed remarkable stability and repeatability (Supplemental Material IV-Fig. S4) with < 3% variation in signal strength after hundreds of cycles. Sensors employing such low-power characteristics will enable the monitoring of almost any explosive threat continuously and in real-time.

## Methods

### Testing procedure

**Figure 10:**
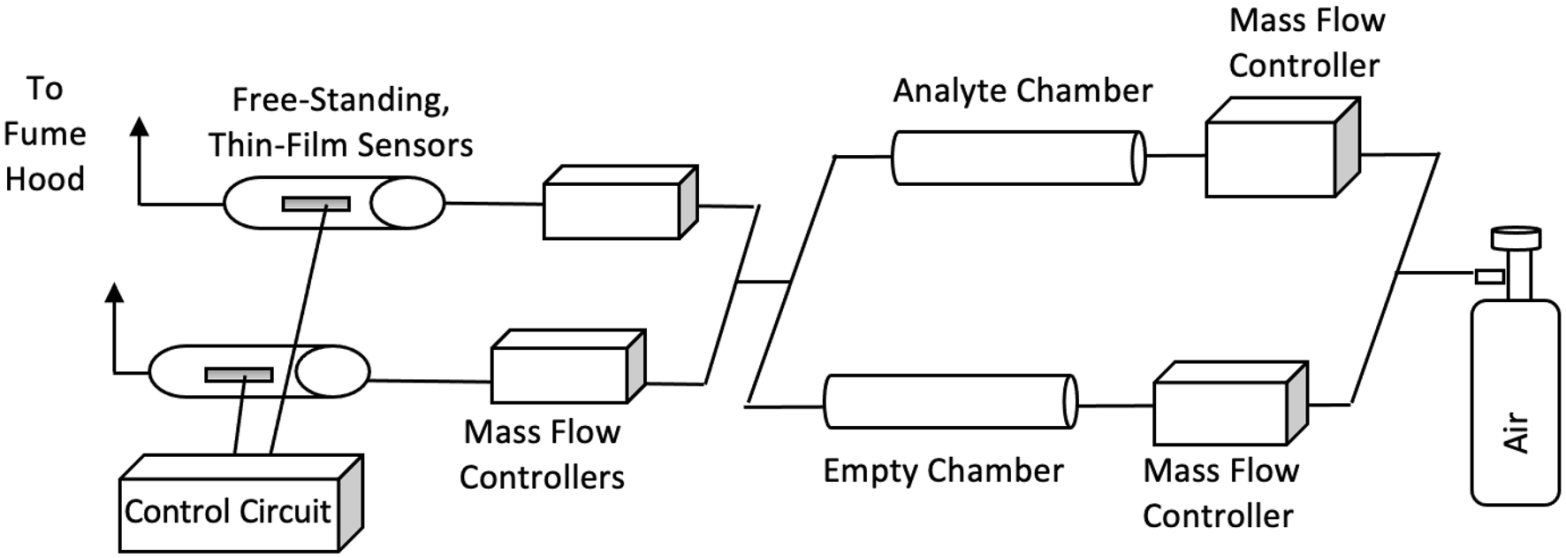
Schematic of apparatus used for testing the free-standing sensors.

As previously mentioned in Ricci et al.^28^, a digital control system was used to supply electrical power to the microheaters such that resistance heating could be accurately controlled using proprietary software. The exact temperature of the microheater can be determined from the temperature coefficient of resistance (TCR) and resting resistance of the palladium microheaters at room temperature. Initially, dry air from a pressurized gas cylinder was split evenly such that identical streams were delivered to catalyst-coated and reference microheaters. Constant volumetric flow was maintained using mass flow controllers (Allicat Scientific Mass Flow Controllers with Flow vision software). At this point the microheater sensors were heated to a predetermined set point temperature, and a power/resistance baseline was established^28^. At the start of a test, the carrier gas was diverted from the empty chamber to the chamber containing solid TATP. Flow was diverted using mass flow controllers so that the volumetric flow rate could be accurately controlled. Explosive vapors were picked up by the carrier gas (dry air) and delivered downstream to the active sensor elements. A schematic of this apparatus is shown in Fig. 10. Our apparatus detects solid explosives with known vapor pressures allowing us to systemically dilute the explosive vapors in real-time. In order to ensure the accuracy of the concentrations delivered by our testing apparatus, responses of an earlier version of our sensor were compared to those independently tested at the Naval Research Laboratory’s TV-Gen vapor generator, which are traceable to NIST standards. The resulting responses were almost identical to those gathered at NRL allowing us to independently confirm the accuracy of our system.

Our thermodynamic sensor platform consists of two separate microheaters: one coated with a metal oxide catalyst, and the other left uncoated, which acts as a reference^27,28^. The reference is used to differentiate between heat effects that arise from catalyst-analyte interactions and those due to hydrodynamic effects including sensible heat effects. By subtracting the reference signal from the catalyst-coated signal, the heat effect due to catalytic decomposition alone is measured. These decomposition reactions can be either exothermic or endothermic and require a change in electrical power to keep the temperature of the two microheaters constant. As these heat effects develop, the software maintains a constant temperature set point by either adding or subtracting electrical power to the catalyst-coated microheater. These electrical power changes required to maintain a constant temperature is in effect, the thermodynamic heat effect associated with these analyte-catalyst interactions^27,28^. The measured power difference is recorded using proprietary software and divided by baseline power; i.e. the power required to maintain the desired temperature setpoint. Here, the electrical power (changes) are reported as a percentage. A typical experiment is run at temperatures as high as 175 °C and requires several minutes to complete, since the sensors were allowed to reach peak response before the analyte supply was shut off. The peak response is the power required to maintain the desired temperature setpoint and reach equilibrium and depends on the response to a specific analyte.

## Supplementary Information

Supplementary Information.

## Data Availability

All data supporting the findings of this study is available within the article and its Supplemental Material.
