# Supplementary material for: Free-standing, thin-film sensors for the trace detection of explosives
Source: Sci Rep. 2021 Mar 23;11:6623. doi: 10.1038/s41598-021-86077-6 (PMC7987993; doi:10.1038/s41598-021-86077-6)
Supplement: Supplementary file 1 — Supplementary Information. [file 41598_2021_86077_MOESM1_ESM.pdf]

Supplemental Material:

Free-Standing, Thin-Film Sensors for the Trace Detection of Explosives

Peter P. Ricci and Otto J. Gregory [*]

Sensors and Surface Technology Partnership, Department of Chemical Engineering,
University of Rhode Island
2 East Alumni Avenue Suite 360 Kingston, RI 02881
[*]Corresponding Author: Dr. Otto Gregory Email: ogregory@uri.edu

Supplemental Material I

URI ESEM Laboratory

Spectrum Report
Monday, November 23, 2020

File: C:\URI Dat...HE539\Pete\New Folder\CuPdthickfilm_5000x_SEI_T002.pgt
Collected: November 23, 2020 13:16:06

Live Time:	30.00	Count Rate:	2848	Dead Time:	22.60 %
Beam Voltage:	20.00	Beam Current:	1.00	Takeoff Angle:	45.00

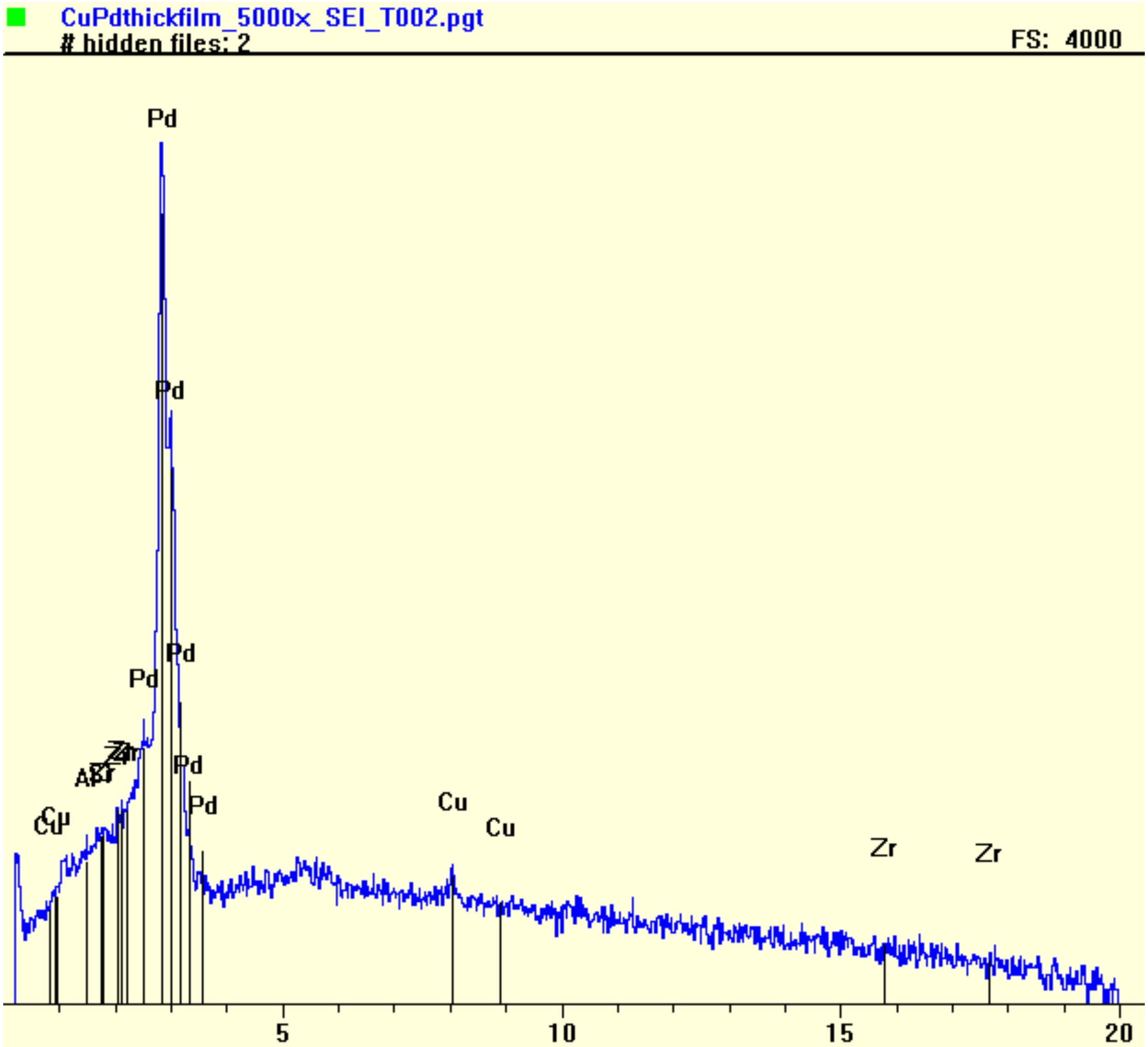

Environmental SEM Lab
4 East Alumni Ave.
Kingston, RI 02881

Tel: 401-874-5828
Fax: 401-782-6422

Supplemental Material II

URI ESEM Laboratory

Spectrum Report
Tuesday, November 24, 2020

File: C:\URI Dat...112420\CuPd_Thickfilm_500C_OOHZ_1000x_bei_SEI_T001.pgt
Collected: November 24, 2020 15:12:26

Live Time:	30.00	Count Rate:	3475	Dead Time:	25.89 %
Beam Voltage:	20.00	Beam Current:	1.00	Takeoff Angle:	45.00

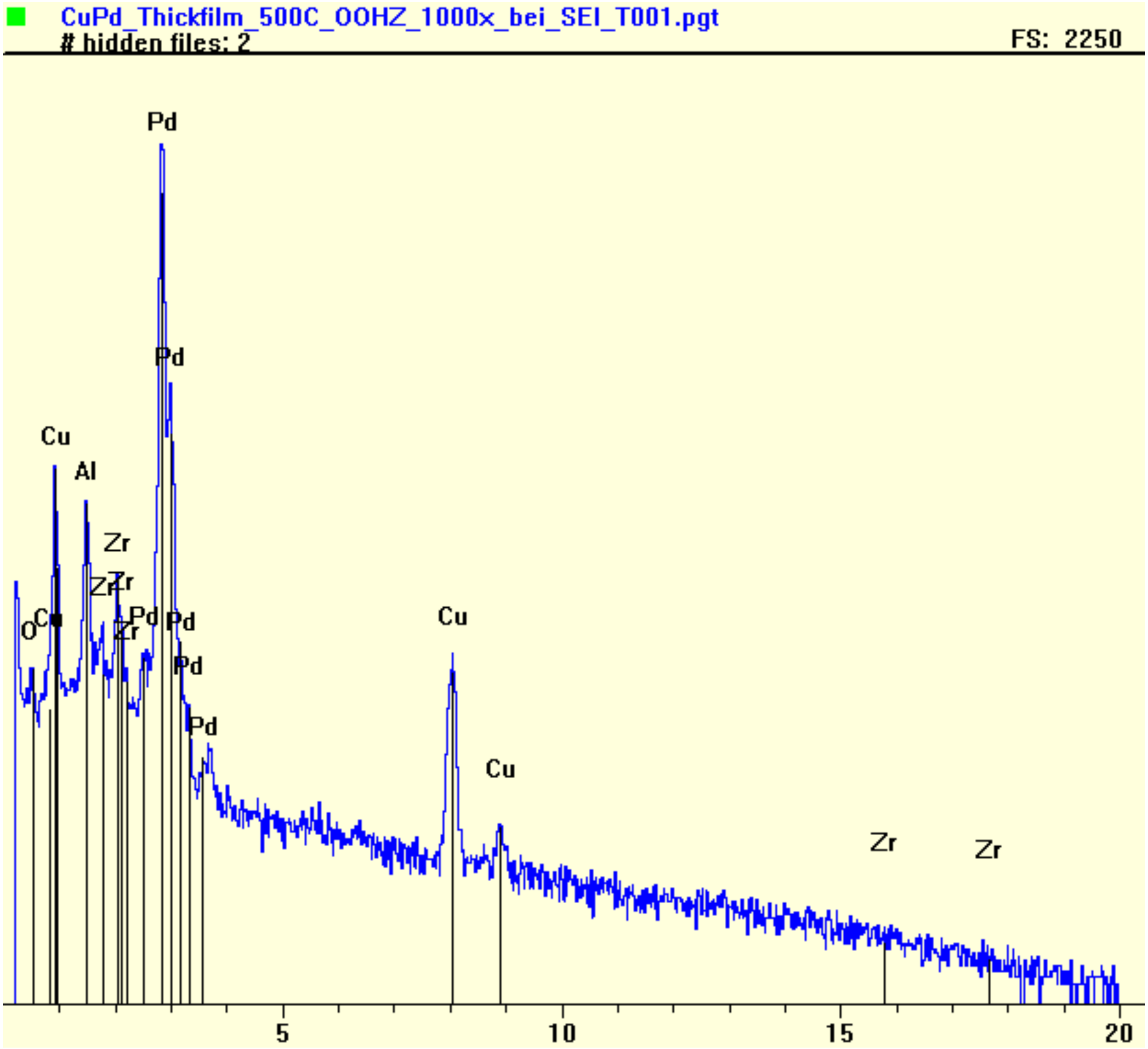

Environmental SEM Lab
4 East Alumni Ave.
Kingston, RI 02881

Tel: 401-874-5828
Fax: 401-782-6422

Supplemental Material III

URI ESEM Laboratory
Image Report
Tuesday, November 24, 2020

File: C:\URI Dat...9\Pete\112420\CuPd_Thickfilm_500C_A3_2500x_bei_SEI.bmp
Collected: November 24, 2020 15:19:45

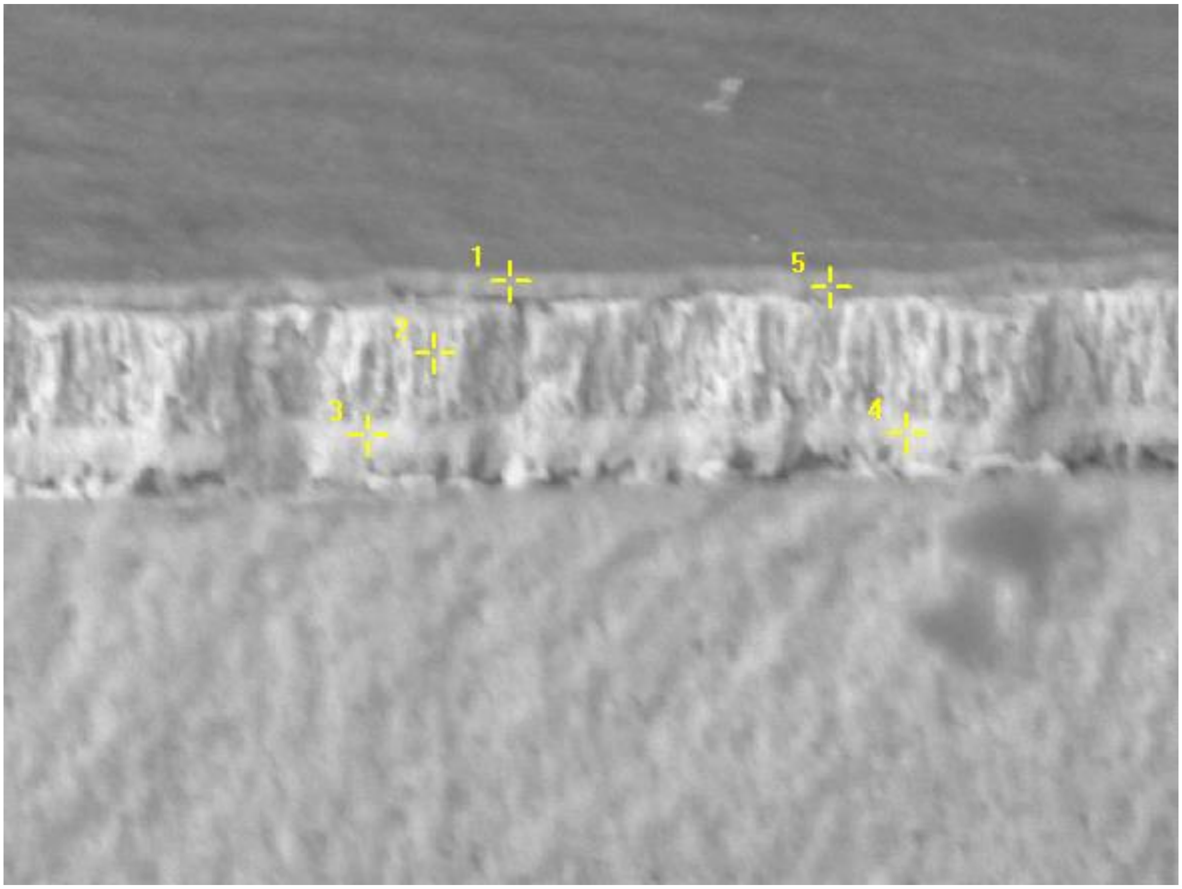

Width(µm): 50.82 Height(µm): 38.12 µm/pixel: 0.050

Scope magnification: 2500X

Environmental SEM Lab
4 East Alumni Ave.
Kingston, RI 02881

Tel: 401-874-5828
Fax: 401-782-6422

URI ESEM Laboratory
Spectrum Report
Tuesday, November 24, 2020

File:	C:\URI Dat...e\112420\CuPd_Thickfilm_500C_A3_2500x_bei_SEI_T001.pgt
Collected:	November 24, 2020 15:21:08

Live Time:	30.00	Count Rate:	2651	Dead Time:	20.30 %
Beam Voltage:	20.00	Beam Current:	1.00	Takeoff Angle:	45.00

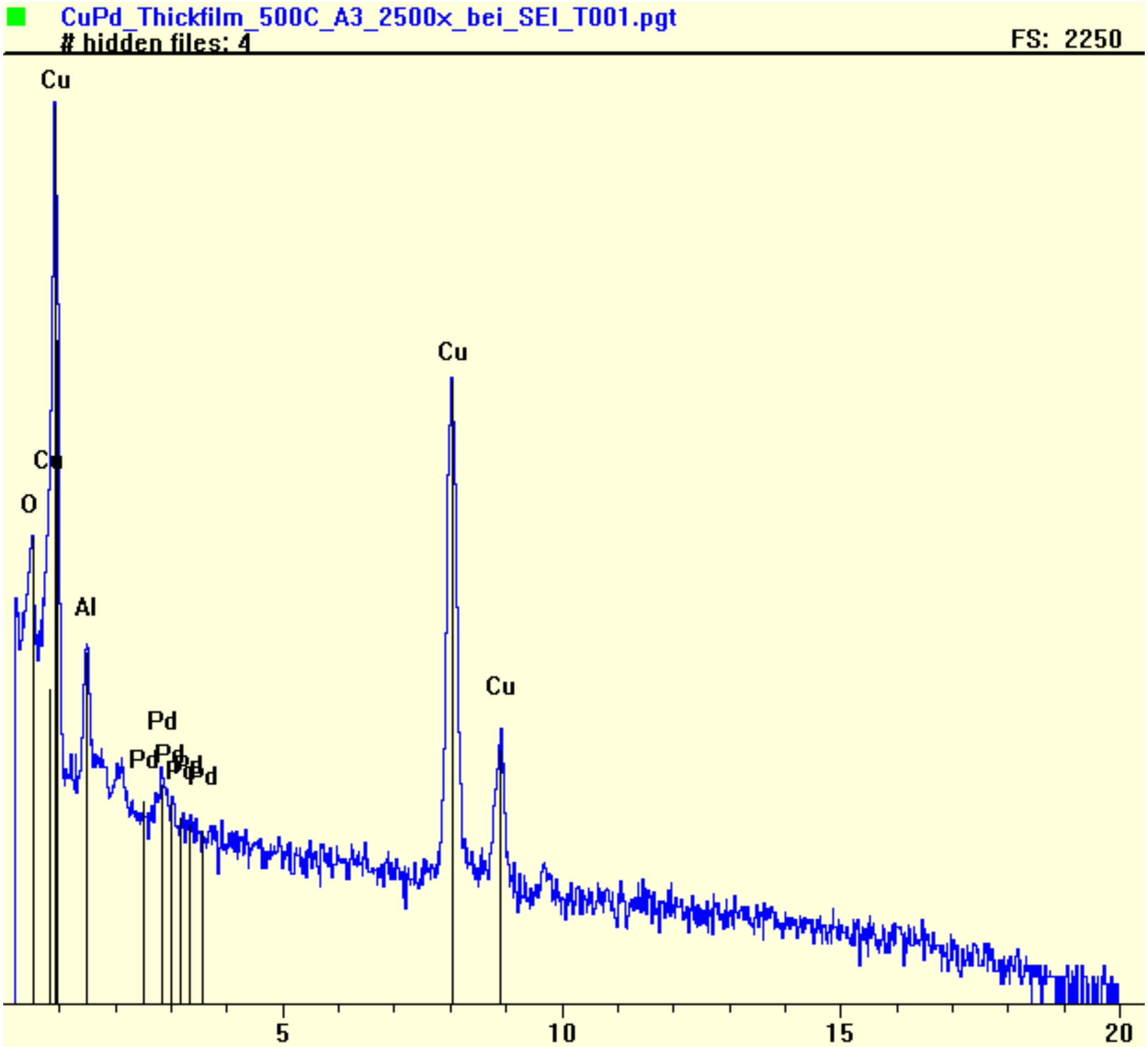

Environmental SEM Lab
4 East Alumni Ave.
Kingston, RI 02881

Tel: 401-874-5828
Fax: 401-782-6422

URI ESEM Laboratory

Spectrum Report

Tuesday, November 24, 2020

File:	C:\URI Dat...e\112420\CuPd_Thickfilm_500C_A3_2500x_bei_SEI_T002.pgt
Collected:	November 24, 2020 15:21:47

Live Time:	30.00	Count Rate:	3013	Dead Time:	23.23 %
Beam Voltage:	20.00	Beam Current:	1.00	Takeoff Angle:	45.00

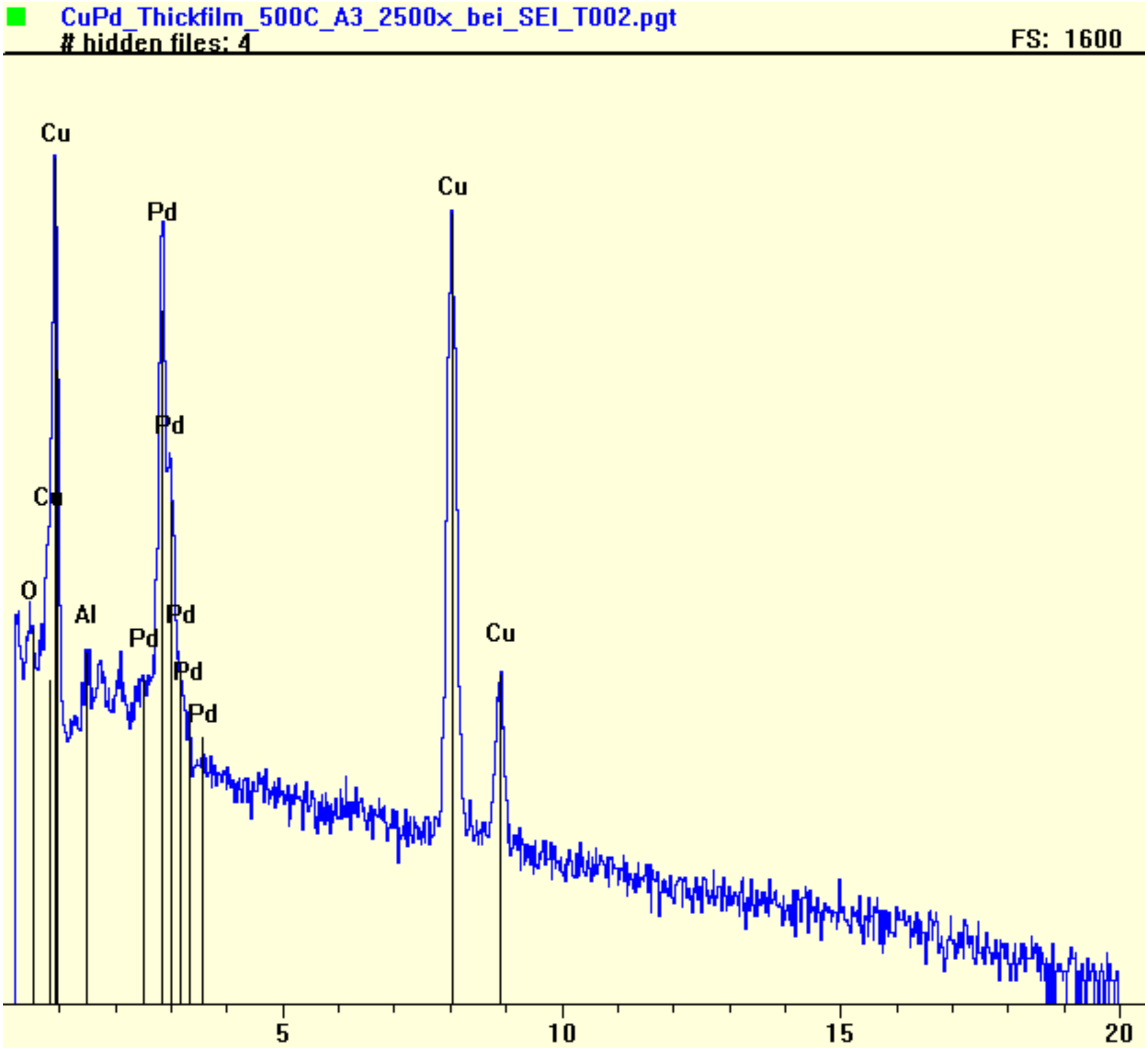

Environmental SEM Lab
4 East Alumni Ave.
Kingston, RI 02881

Tel: 401-874-5828
Fax: 401-782-6422

Supplemental Material IV

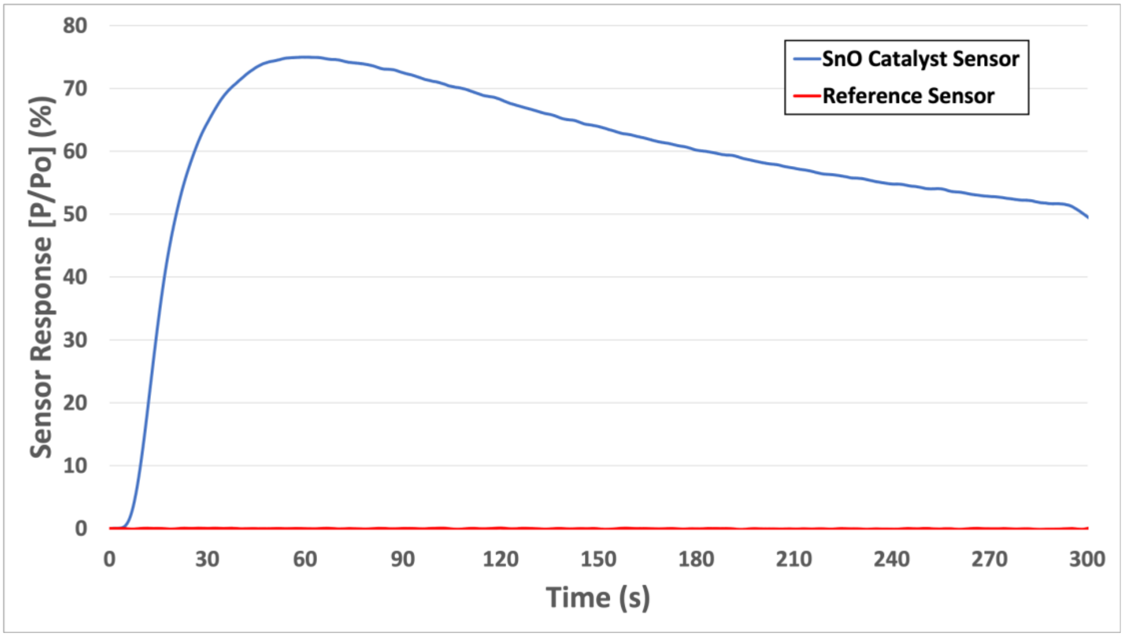

Figure S1: Original testing data showing the responses of the SnO^{1+} coated free-standing sensor and the reference free-standing sensor to 20ppm TATP. The reference sensor does not employ a catalyst coating and thus is unresponsive to the analyte. Additionally, the natively grown CuO, as a result of the fabrication process, is critically thin and thus is also not shown to respond to the analyte.

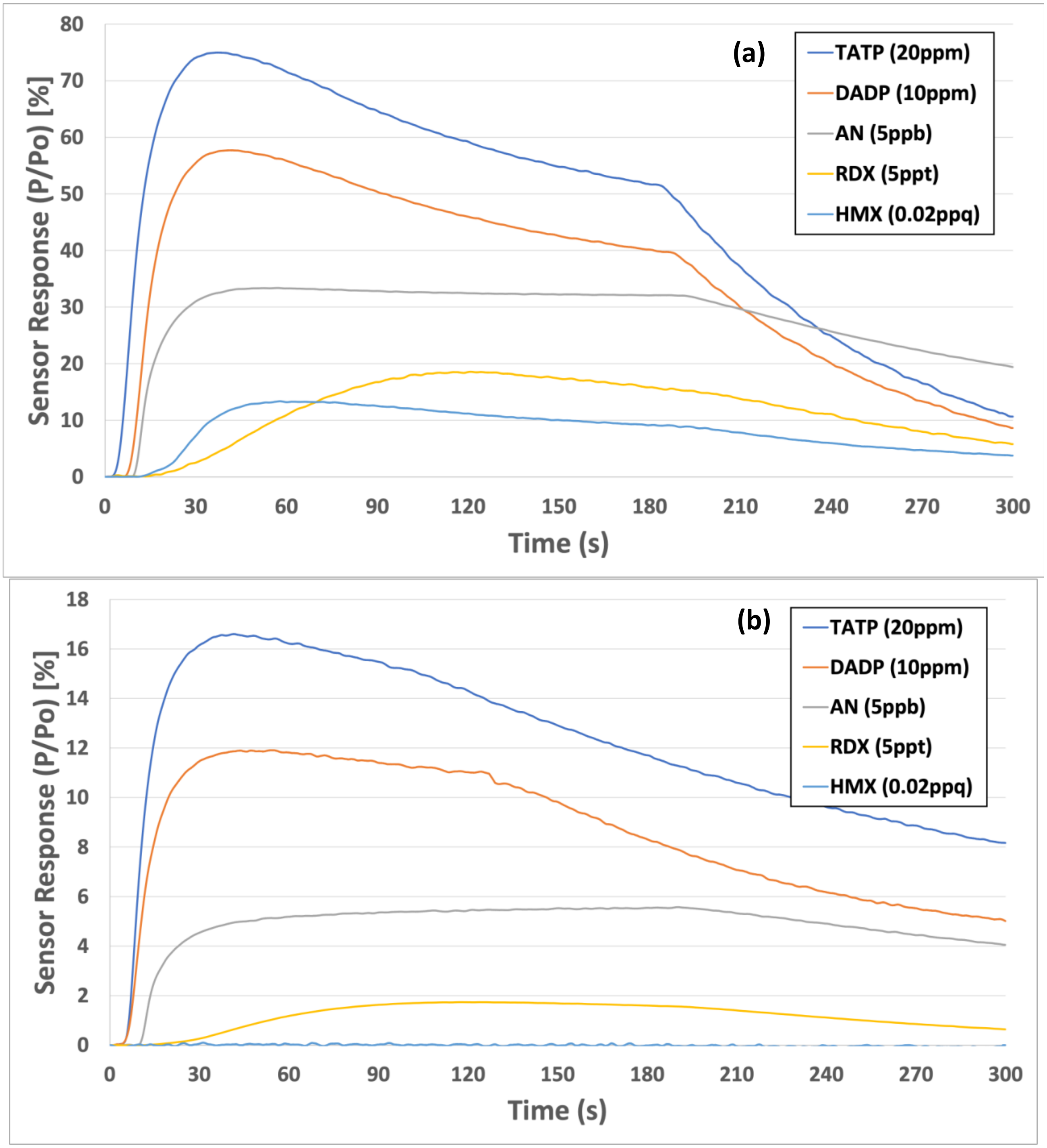

Figure S2: Original testing data for the free-standing sensor (a) and the 8µm YSZ sensor (b) shown in Figure 9. The variations in overall sensor response, response time and recovery time are attributed to different concentrations and mass transport variations for each explosive.

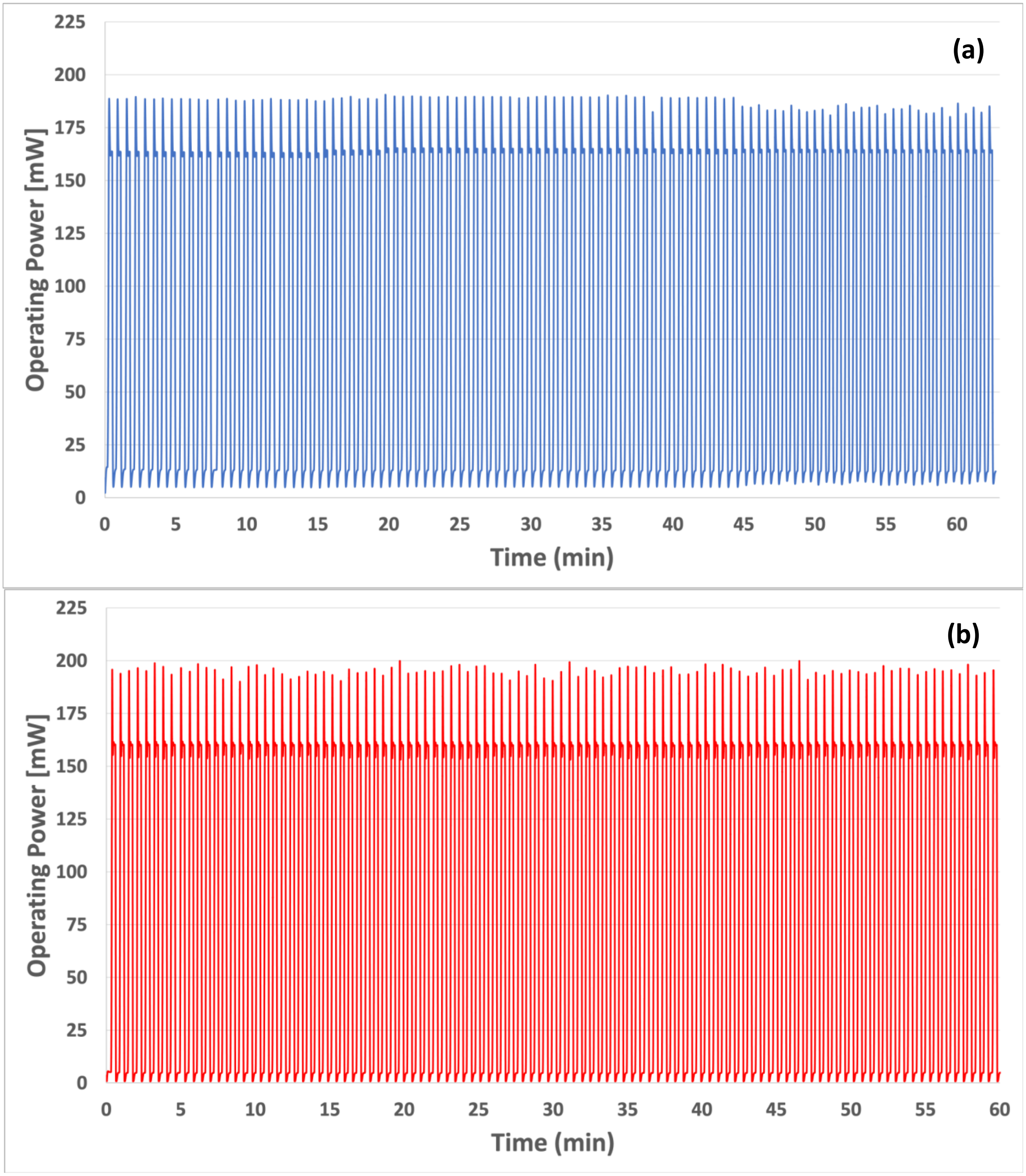

Figure S3: Thermal cycling data (~100 cycles) for the catalyst coated sensor (a) and the reference sensor (b) in air (50% RH). Here, the sensors were cycled from room temperature (25°C) to the operating temperature (175°C) where the power required to heat the sensors to 175°C is ~160mW. Each cycle is shown to overshoot before establishing the operating power baseline.

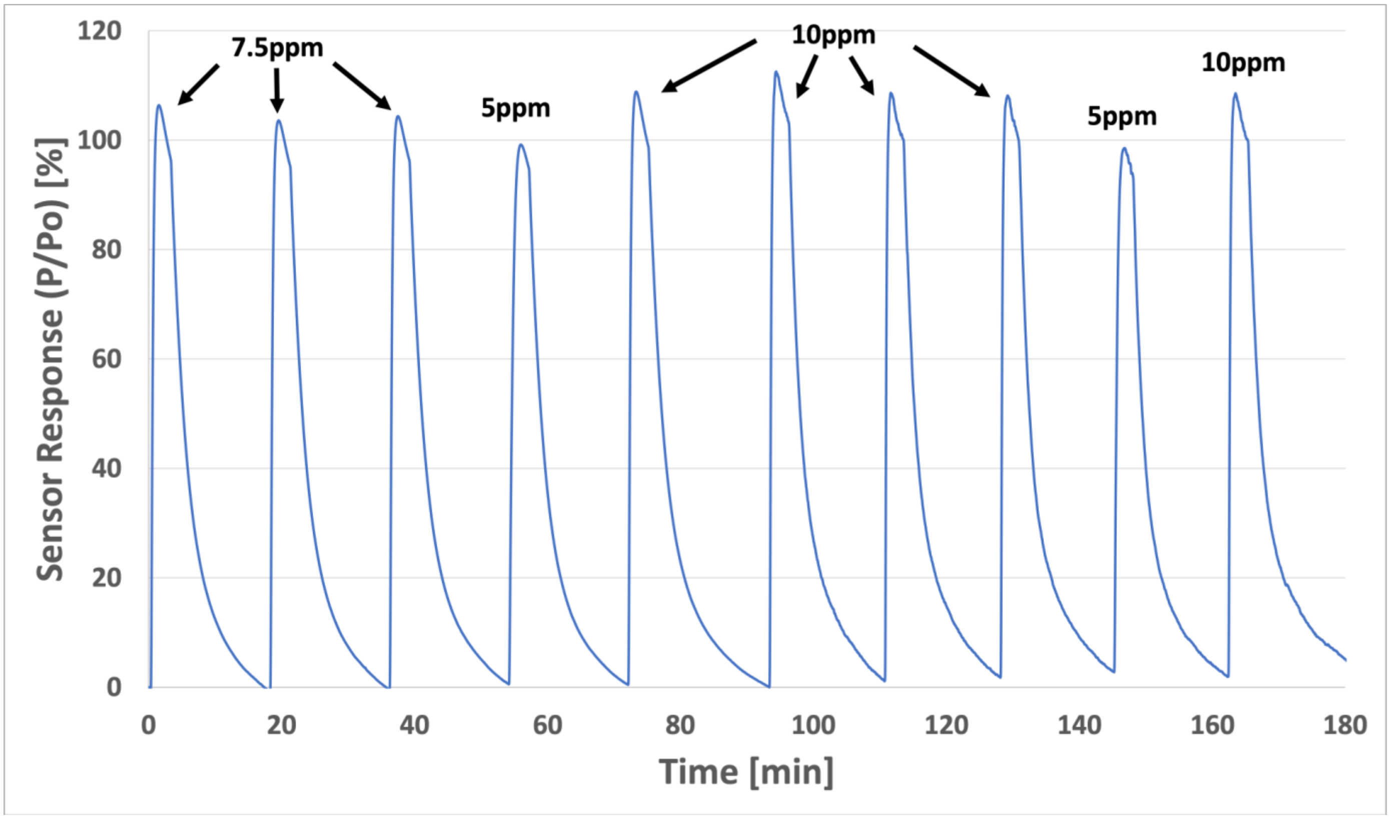

Figure S4: Acetone calibration data for the catalyst coated free-standing sensor. Here, the free-standing sensor is exposed to three distinct concentrations of acetone (5ppm, 7.5ppm, and 10ppm) to determine stability and repeatability prior to testing with explosives.
